# Short-term stock market price trend prediction using a comprehensive deep learning system

**DOI:** 10.1186/s40537-020-00333-6

**Published:** 2020-08-28

**Authors:** Jingyi Shen, M. Omair Shafiq

**Affiliations:** School of Information Technology, Carleton University, Ottawa, ON Canada

**Keywords:** Prediction, Deep learning, Stock market trend, Feature engineering

## Abstract

In the era of big data, deep learning for predicting stock market prices and trends has become even more popular than before. We collected 2 years of data from Chinese stock market and proposed a comprehensive customization of feature engineering and deep learning-based model for predicting price trend of stock markets. The proposed solution is comprehensive as it includes pre-processing of the stock market dataset, utilization of multiple feature engineering techniques, combined with a customized deep learning based system for stock market price trend prediction. We conducted comprehensive evaluations on frequently used machine learning models and conclude that our proposed solution outperforms due to the comprehensive feature engineering that we built. The system achieves overall high accuracy for stock market trend prediction. With the detailed design and evaluation of prediction term lengths, feature engineering, and data pre-processing methods, this work contributes to the stock analysis research community both in the financial and technical domains.

## Introduction

Stock market is one of the major fields that investors are dedicated to, thus stock market price trend prediction is always a hot topic for researchers from both financial and technical domains. In this research, our objective is to build a state-of-art prediction model for price trend prediction, which focuses on short-term price trend prediction.

As concluded by Fama in [26], financial time series prediction is known to be a notoriously difficult task due to the generally accepted, semi-strong form of market efficiency and the high level of noise. Back in 2003, Wang et al. in [44] already applied artificial neural networks on stock market price prediction and focused on volume, as a specific feature of stock market. One of the key findings by them was that the volume was not found to be effective in improving the forecasting performance on the datasets they used, which was S&P 500 and DJI. Ince and Trafalis in [15] targeted short-term forecasting and applied support vector machine (SVM) model on the stock price prediction. Their main contribution is performing a comparison between multi-layer perceptron (MLP) and SVM then found that most of the scenarios SVM outperformed MLP, while the result was also affected by different trading strategies. In the meantime, researchers from financial domains were applying conventional statistical methods and signal processing techniques on analyzing stock market data.

The optimization techniques, such as principal component analysis (PCA) were also applied in short-term stock price prediction [22]. During the years, researchers are not only focused on stock price-related analysis but also tried to analyze stock market transactions such as volume burst risks, which expands the stock market analysis research domain broader and indicates this research domain still has high potential [39]. As the artificial intelligence techniques evolved in recent years, many proposed solutions attempted to combine machine learning and deep learning techniques based on previous approaches, and then proposed new metrics that serve as training features such as Liu and Wang [23]. This type of previous works belongs to the feature engineering domain and can be considered as the inspiration of feature extension ideas in our research. Liu et al. in [24] proposed a convolutional neural network (CNN) as well as a long short-term memory (LSTM) neural network based model to analyze different quantitative strategies in stock markets. The CNN serves for the stock selection strategy, automatically extracts features based on quantitative data, then follows an LSTM to preserve the time-series features for improving profits.

The latest work also proposes a similar hybrid neural network architecture, integrating a convolutional neural network with a bidirectional long short-term memory to predict the stock market index [4]. While the researchers frequently proposed different neural network solution architectures, it brought further discussions about the topic if the high cost of training such models is worth the result or not.

There are three key contributions of our work (1) a new dataset extracted and cleansed (2) a comprehensive feature engineering, and (3) a customized long short-term memory (LSTM) based deep learning model.

We have built the dataset by ourselves from the data source as an open-sourced data API called Tushare [43]. The novelty of our proposed solution is that we proposed a feature engineering along with a fine-tuned system instead of just an LSTM model only. We observe from the previous works and find the gaps and proposed a solution architecture with a comprehensive feature engineering procedure before training the prediction model. With the success of feature extension method collaborating with recursive feature elimination algorithms, it opens doors for many other machine learning algorithms to achieve high accuracy scores for short-term price trend prediction. It proved the effectiveness of our proposed feature extension as feature engineering. We further introduced our customized LSTM model and further improved the prediction scores in all the evaluation metrics. The proposed solution outperformed the machine learning and deep learning-based models in similar previous works.

The remainder of this paper is organized as follows. “Survey of related works” section describes the survey of related works. “The dataset” section provides details on the data that we extracted from the public data sources and the dataset prepared. “Methods” section presents the research problems, methods, and design of the proposed solution. Detailed technical design with algorithms and how the model implemented are also included in this section. “Results” section presents comprehensive results and evaluation of our proposed model, and by comparing it with the models used in most of the related works. “Discussion” section provides a discussion and comparison of the results. “Conclusion” section presents the conclusion. This research paper has been built based on Shen [36].

## Survey of related works

In this section, we discuss related works. We reviewed the related work in two different domains: technical and financial, respectively.

Kim and Han in [19] built a model as a combination of artificial neural networks (ANN) and genetic algorithms (GAs) with discretization of features for predicting stock price index. The data used in their study include the technical indicators as well as the direction of change in the daily Korea stock price index (KOSPI). They used the data containing samples of 2928 trading days, ranging from January 1989 to December 1998, and give their selected features and formulas. They also applied optimization of feature discretization, as a technique that is similar to dimensionality reduction. The strengths of their work are that they introduced GA to optimize the ANN. First, the amount of input features and processing elements in the hidden layer are 12 and not adjustable. Another limitation is in the learning process of ANN, and the authors only focused on two factors in optimization. While they still believed that GA has great potential for feature discretization optimization. Our initialized feature pool refers to the selected features. Qiu and Song in [34] also presented a solution to predict the direction of the Japanese stock market based on an optimized artificial neural network model. In this work, authors utilize genetic algorithms together with artificial neural network based models, and name it as a hybrid GA-ANN model.

Piramuthu in [33] conducted a thorough evaluation of different feature selection methods for data mining applications. He used for datasets, which were credit approval data, loan defaults data, web traffic data, tam, and kiang data, and compared how different feature selection methods optimized decision tree performance. The feature selection methods he compared included probabilistic distance measure: the Bhattacharyya measure, the Matusita measure, the divergence measure, the Mahalanobis distance measure, and the Patrick-Fisher measure. For inter-class distance measures: the Minkowski distance measure, city block distance measure, Euclidean distance measure, the Chebychev distance measure, and the nonlinear (Parzen and hyper-spherical kernel) distance measure. The strength of this paper is that the author evaluated both probabilistic distance-based and several inter-class feature selection methods. Besides, the author performed the evaluation based on different datasets, which reinforced the strength of this paper. However, the evaluation algorithm was a decision tree only. We cannot conclude if the feature selection methods will still perform the same on a larger dataset or a more complex model.

Hassan and Nath in [9] applied the Hidden Markov Model (HMM) on the stock market forecasting on stock prices of four different Airlines. They reduce states of the model into four states: the opening price, closing price, the highest price, and the lowest price. The strong point of this paper is that the approach does not need expert knowledge to build a prediction model. While this work is limited within the industry of Airlines and evaluated on a very small dataset, it may not lead to a prediction model with generality. One of the approaches in stock market prediction related works could be exploited to do the comparison work. The authors selected a maximum 2 years as the date range of training and testing dataset, which provided us a date range reference for our evaluation part.

Lei in [21] exploited Wavelet Neural Network (WNN) to predict stock price trends. The author also applied Rough Set (RS) for attribute reduction as an optimization. Rough Set was utilized to reduce the stock price trend feature dimensions. It was also used to determine the structure of the Wavelet Neural Network. The dataset of this work consists of five well-known stock market indices, i.e., (1) SSE Composite Index (China), (2) CSI 300 Index (China), (3) All Ordinaries Index (Australian), (4) Nikkei 225 Index (Japan), and (5) Dow Jones Index (USA). Evaluation of the model was based on different stock market indices, and the result was convincing with generality. By using Rough Set for optimizing the feature dimension before processing reduces the computational complexity. However, the author only stressed the parameter adjustment in the discussion part but did not specify the weakness of the model itself. Meanwhile, we also found that the evaluations were performed on indices, the same model may not have the same performance if applied on a specific stock.

Lee in [20] used the support vector machine (SVM) along with a hybrid feature selection method to carry out prediction of stock trends. The dataset in this research is a sub dataset of NASDAQ Index in Taiwan Economic Journal Database (TEJD) in 2008. The feature selection part was using a hybrid method, supported sequential forward search (SSFS) played the role of the wrapper. Another advantage of this work is that they designed a detailed procedure of parameter adjustment with performance under different parameter values. The clear structure of the feature selection model is also heuristic to the primary stage of model structuring. One of the limitations was that the performance of SVM was compared to back-propagation neural network (BPNN) only and did not compare to the other machine learning algorithms.

Sirignano and Cont leveraged a deep learning solution trained on a universal feature set of financial markets in [40]. The dataset used included buy and sell records of all transactions, and cancellations of orders for approximately 1000 NASDAQ stocks through the order book of the stock exchange. The NN consists of three layers with LSTM units and a feed-forward layer with rectified linear units (ReLUs) at last, with stochastic gradient descent (SGD) algorithm as an optimization. Their universal model was able to generalize and cover the stocks other than the ones in the training data. Though they mentioned the advantages of a universal model, the training cost was still expensive. Meanwhile, due to the inexplicit programming of the deep learning algorithm, it is unclear that if there are useless features contaminated when feeding the data into the model. Authors found out that it would have been better if they performed feature selection part before training the model and found it as an effective way to reduce the computational complexity.

Ni et al. in [30] predicted stock price trends by exploiting SVM and performed fractal feature selection for optimization. The dataset they used is the Shanghai Stock Exchange Composite Index (SSECI), with 19 technical indicators as features. Before processing the data, they optimized the input data by performing feature selection. When finding the best parameter combination, they also used a grid search method, which is k cross-validation. Besides, the evaluation of different feature selection methods is also comprehensive. As the authors mentioned in their conclusion part, they only considered the technical indicators but not macro and micro factors in the financial domain. The source of datasets that the authors used was similar to our dataset, which makes their evaluation results useful to our research. They also mentioned a method called k cross-validation when testing hyper-parameter combinations.

McNally et al. in [27] leveraged RNN and LSTM on predicting the price of Bitcoin, optimized by using the Boruta algorithm for feature engineering part, and it works similarly to the random forest classifier. Besides feature selection, they also used Bayesian optimization to select LSTM parameters. The Bitcoin dataset ranged from the 19th of August 2013 to 19th of July 2016. Used multiple optimization methods to improve the performance of deep learning methods. The primary problem of their work is overfitting. The research problem of predicting Bitcoin price trend has some similarities with stock market price prediction. Hidden features and noises embedded in the price data are threats of this work. The authors treated the research question as a time sequence problem. The best part of this paper is the feature engineering and optimization part; we could replicate the methods they exploited in our data pre-processing.

Weng et al. in [45] focused on short-term stock price prediction by using ensemble methods of four well-known machine learning models. The dataset for this research is five sets of data. They obtained these datasets from three open-sourced APIs and an R package named TTR. The machine learning models they used are (1) neural network regression ensemble (NNRE), (2) a Random Forest with unpruned regression trees as base learners (RFR), (3) AdaBoost with unpruned regression trees as base learners (BRT) and (4) a support vector regression ensemble (SVRE). A thorough study of ensemble methods specified for short-term stock price prediction. With background knowledge, the authors selected eight technical indicators in this study then performed a thoughtful evaluation of five datasets. The primary contribution of this paper is that they developed a platform for investors using R, which does not need users to input their own data but call API to fetch the data from online source straightforward. From the research perspective, they only evaluated the prediction of the price for 1 up to 10 days ahead but did not evaluate longer terms than two trading weeks or a shorter term than 1 day. The primary limitation of their research was that they only analyzed 20 U.S.-based stocks, the model might not be generalized to other stock market or need further revalidation to see if it suffered from overfitting problems.

Kara et al. in [17] also exploited ANN and SVM in predicting the movement of stock price index. The data set they used covers a time period from January 2, 1997, to December 31, 2007, of the Istanbul Stock Exchange. The primary strength of this work is its detailed record of parameter adjustment procedures. While the weaknesses of this work are that neither the technical indicator nor the model structure has novelty, and the authors did not explain how their model performed better than other models in previous works. Thus, more validation works on other datasets would help. They explained how ANN and SVM work with stock market features, also recorded the parameter adjustment. The implementation part of our research could benefit from this previous work.

Jeon et al. in [16] performed research on millisecond interval-based big dataset by using pattern graph tracking to complete stock price prediction tasks. The dataset they used is a millisecond interval-based big dataset of historical stock data from KOSCOM, from August 2014 to October 2014, 10G–15G capacity. The author applied Euclidean distance, Dynamic Time Warping (DTW) for pattern recognition. For feature selection, they used stepwise regression. The authors completed the prediction task by ANN and Hadoop and RHive for big data processing. The “Results” section is based on the result processed by a combination of SAX and Jaro–Winkler distance. Before processing the data, they generated aggregated data at 5-min intervals from discrete data. The primary strength of this work is the explicit structure of the whole implementation procedure. While they exploited a relatively old model, another weakness is the overall time span of the training dataset is extremely short. It is difficult to access the millisecond interval-based data in real life, so the model is not as practical as a daily based data model.

Huang et al. in [12] applied a fuzzy-GA model to complete the stock selection task. They used the key stocks of the 200 largest market capitalization listed as the investment universe in the Taiwan Stock Exchange. Besides, the yearly financial statement data and the stock returns were taken from the Taiwan Economic Journal (TEJ) database at www.tej.com.tw/ for the time period from year 1995 to year 2009. They conducted the fuzzy membership function with model parameters optimized with GA and extracted features for optimizing stock scoring. The authors proposed an optimized model for selection and scoring of stocks. Different from the prediction model, the authors more focused on stock rankings, selection, and performance evaluation. Their structure is more practical among investors. But in the model validation part, they did not compare the model with existed algorithms but the statistics of the benchmark, which made it challenging to identify if GA would outperform other algorithms.

Fischer and Krauss in [5] applied long short-term memory (LSTM) on financial market prediction. The dataset they used is S&P 500 index constituents from Thomson Reuters. They obtained all month-end constituent lists for the S&P 500 from Dec 1989 to Sep 2015, then consolidated the lists into a binary matrix to eliminate survivor bias. The authors also used RMSprop as an optimizer, which is a mini-batch version of rprop. The primary strength of this work is that the authors used the latest deep learning technique to perform predictions. They relied on the LSTM technique, lack of background knowledge in the financial domain. Although the LSTM outperformed the standard DNN and logistic regression algorithms, while the author did not mention the effort to train an LSTM with long-time dependencies.

Tsai and Hsiao in [42] proposed a solution as a combination of different feature selection methods for prediction of stocks. They used Taiwan Economic Journal (TEJ) database as data source. The data used in their analysis was from year 2000 to 2007. In their work, they used a sliding window method and combined it with multi layer perceptron (MLP) based artificial neural networks with back propagation, as their prediction model. In their work, they also applied principal component analysis (PCA) for dimensionality reduction, genetic algorithms (GA) and the classification and regression trees (CART) to select important features. They did not just rely on technical indices only. Instead, they also included both fundamental and macroeconomic indices in their analysis. The authors also reported a comparison on feature selection methods. The validation part was done by combining the model performance stats with statistical analysis.

Pimenta et al. in [32] leveraged an automated investing method by using multi-objective genetic programming and applied it in the stock market. The dataset was obtained from Brazilian stock exchange market (BOVESPA), and the primary techniques they exploited were a combination of multi-objective optimization, genetic programming, and technical trading rules. For optimization, they leveraged genetic programming (GP) to optimize decision rules. The novelty of this paper was in the evaluation part. They included a historical period, which was a critical moment of Brazilian politics and economics when performing validation. This approach reinforced the generalization strength of their proposed model. When selecting the sub-dataset for evaluation, they also set criteria to ensure more asset liquidity. While the baseline of the comparison was too basic and fundamental, and the authors did not perform any comparison with other existing models.

Huang and Tsai in [13] conducted a filter-based feature selection assembled with a hybrid self-organizing feature map (SOFM) support vector regression (SVR) model to forecast Taiwan index futures (FITX) trend. They divided the training samples into clusters to marginally improve the training efficiency. The authors proposed a comprehensive model, which was a combination of two novel machine learning techniques in stock market analysis. Besides, the optimizer of feature selection was also applied before the data processing to improve the prediction accuracy and reduce the computational complexity of processing daily stock index data. Though they optimized the feature selection part and split the sample data into small clusters, it was already strenuous to train daily stock index data of this model. It would be difficult for this model to predict trading activities in shorter time intervals since the data volume would be increased drastically. Moreover, the evaluation is not strong enough since they set a single SVR model as a baseline, but did not compare the performance with other previous works, which caused difficulty for future researchers to identify the advantages of SOFM-SVR model why it outperforms other algorithms.

Thakur and Kumar in [41] also developed a hybrid financial trading support system by exploiting multi-category classifiers and random forest (RAF). They conducted their research on stock indices from NASDAQ, DOW JONES, S&P 500, NIFTY 50, and NIFTY BANK. The authors proposed a hybrid model combined random forest (RF) algorithms with a weighted multicategory generalized eigenvalue support vector machine (WMGEPSVM) to generate “Buy/Hold/Sell” signals. Before processing the data, they used Random Forest (RF) for feature pruning. The authors proposed a practical model designed for real-life investment activities, which could generate three basic signals for investors to refer to. They also performed a thorough comparison of related algorithms. While they did not mention the time and computational complexity of their works. Meanwhile, the unignorable issue of their work was the lack of financial domain knowledge background. The investors regard the indices data as one of the attributes but could not take the signal from indices to operate a specific stock straightforward.

Hsu in [11] assembled feature selection with a back propagation neural network (BNN) combined with genetic programming to predict the stock/futures price. The dataset in this research was obtained from Taiwan Stock Exchange Corporation (TWSE). The authors have introduced the description of the background knowledge in detail. While the weakness of their work is that it is a lack of data set description. This is a combination of the model proposed by other previous works. Though we did not see the novelty of this work, we can still conclude that the genetic programming (GP) algorithm is admitted in stock market research domain. To reinforce the validation strengths, it would be good to consider adding GP models into evaluation if the model is predicting a specific price.

Hafezi et al. in [7] built a bat-neural network multi-agent system (BN-NMAS) to predict stock price. The dataset was obtained from the Deutsche bundes-bank. They also applied the Bat algorithm (BA) for optimizing neural network weights. The authors illustrated their overall structure and logic of system design in clear flowcharts. While there were very few previous works that had performed on DAX data, it would be difficult to recognize if the model they proposed still has the generality if migrated on other datasets. The system design and feature selection logic are fascinating, which worth referring to. Their findings in optimization algorithms are also valuable for the research in the stock market price prediction research domain. It is worth trying the Bat algorithm (BA) when constructing neural network models.

Long et al. in [25] conducted a deep learning approach to predict the stock price movement. The dataset they used is the Chinese stock market index CSI 300. For predicting the stock price movement, they constructed a multi-filter neural network (MFNN) with stochastic gradient descent (SGD) and back propagation optimizer for learning NN parameters. The strength of this paper is that the authors exploited a novel model with a hybrid model constructed by different kinds of neural networks, it provides an inspiration for constructing hybrid neural network structures.

Atsalakis and Valavanis in [1] proposed a solution of a neuro-fuzzy system, which is composed of controller named as Adaptive Neuro Fuzzy Inference System (ANFIS), to achieve short-term stock price trend prediction. The noticeable strength of this work is the evaluation part. Not only did they compare their proposed system with the popular data models, but also compared with investment strategies. While the weakness that we found from their proposed solution is that their solution architecture is lack of optimization part, which might limit their model performance. Since our proposed solution is also focusing on short-term stock price trend prediction, this work is heuristic for our system design. Meanwhile, by comparing with the popular trading strategies from investors, their work inspired us to compare the strategies used by investors with techniques used by researchers.

Nekoeiqachkanloo et al. in [29] proposed a system with two different approaches for stock investment. The strengths of their proposed solution are obvious. First, it is a comprehensive system that consists of data pre-processing and two different algorithms to suggest the best investment portions. Second, the system also embedded with a forecasting component, which also retains the features of the time series. Last but not least, their input features are a mix of fundamental features and technical indices that aim to fill in the gap between the financial domain and technical domain. However, their work has a weakness in the evaluation part. Instead of evaluating the proposed system on a large dataset, they chose 25 well-known stocks. There is a high possibility that the well-known stocks might potentially share some common hidden features.

As another related latest work, Idrees et al. [14] published a time series-based prediction approach for the volatility of the stock market. ARIMA is not a new approach in the time series prediction research domain. Their work is more focusing on the feature engineering side. Before feeding the features into ARIMA models, they designed three steps for feature engineering: Analyze the time series, identify if the time series is stationary or not, perform estimation by plot ACF and PACF charts and look for parameters. The only weakness of their proposed solution is that the authors did not perform any customization on the existing ARIMA model, which might limit the system performance to be improved.

One of the main weaknesses found in the related works is limited data-preprocessing mechanisms built and used. Technical works mostly tend to focus on building prediction models. When they select the features, they list all the features mentioned in previous works and go through the feature selection algorithm then select the best-voted features. Related works in the investment domain have shown more interest in behavior analysis, such as how herding behaviors affect the stock performance, or how the percentage of inside directors hold the firm’s common stock affects the performance of a certain stock. These behaviors often need a pre-processing procedure of standard technical indices and investment experience to recognize.

In the related works, often a thorough statistical analysis is performed based on a special dataset and conclude new features rather than performing feature selections. Some data, such as the percentage of a certain index fluctuation has been proven to be effective on stock performance. We believe that by extracting new features from data, then combining such features with existed common technical indices will significantly benefit the existing and well-tested prediction models.

## The dataset

This section details the data that was extracted from the public data sources, and the final dataset that was prepared. Stock market-related data are diverse, so we first compared the related works from the survey of financial research works in stock market data analysis to specify the data collection directions. After collecting the data, we defined a data structure of the dataset. Given below, we describe the dataset in detail, including the data structure, and data tables in each category of data with the segment definitions.

### Description of our dataset

In this section, we will describe the dataset in detail. This dataset consists of 3558 stocks from the Chinese stock market. Besides the daily price data, daily fundamental data of each stock ID, we also collected the suspending and resuming history, top 10 shareholders, etc. We list two reasons that we choose 2 years as the time span of this dataset: (1) most of the investors perform stock market price trend analysis using the data within the latest 2 years, (2) using more recent data would benefit the analysis result. We collected data through the open-sourced API, namely Tushare [43], mean-while we also leveraged a web-scraping technique to collect data from Sina Finance web pages, SWS Research website.

### Data structure

**Fig. 1:**
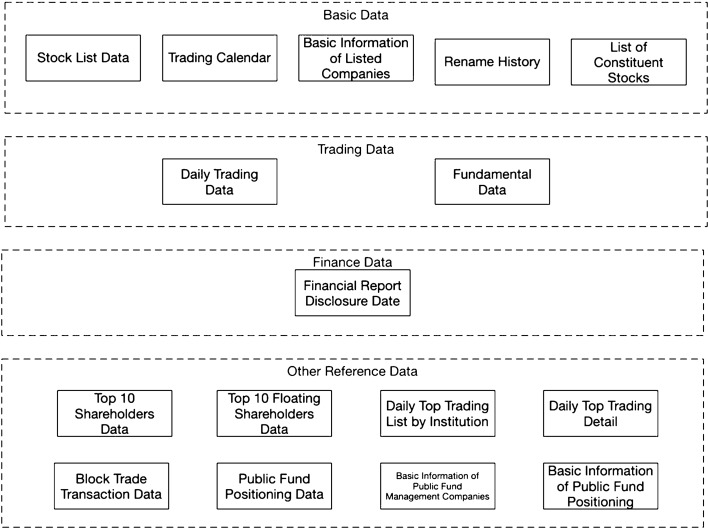
Data structure for the extracted dataset

Figure 1 illustrates all the data tables in the dataset. We collected four categories of data in this dataset: (1) basic data, (2) trading data, (3) finance data, and (4) other reference data. All the data tables can be linked to each other by a common field called “Stock ID” It is a unique stock identifier registered in the Chinese Stock market. Table 1 shows an overview of the dataset.

**Table 1:** Dataset overview table with different categories and subsets of fields

Data table name	Category	Field
Stock list	Basic data	Stock ID, Stock name, Geographic info, Industry, Full name, English name, Market type, Stock exchange ID, Currency, List status, List date, Delist date, If the stock is HS constituent
Trading calendar	Basic data	Stock exchange ID, Calendar date, If the date is open for trading, Pervious trading date
Basic information of listed companies	Basic data	Stock ID, Stock exchange ID, Corporate representative, General manager, Secretary, Authorized capital, Registration date, Province, City, Introduction, Website, Email, Office address, Number of employees, Main business, Business scope
Renamed history	Basic data	Stock ID, Stock name, Start date, End date, Announcement date, Rename reason
Constituent stock information	Basic data	Stock ID, Constituent type, Included date, Excluded date, If the stock is new
Daily trading data	Trading data	Stock ID, Trading date, Opening price, Highest price, Lowest price, Closing price, Previous closing price, Price change, Price change percentage, Volume, Amount
Fundamental data	Trading data	Stock ID, Trading date, Closing price, Turnover rate, Free turnover rate, Volume ratio, Price-to-earning ratio, Price-to-earning ratio TTM, Price-to-book ratio, Price-to-sales ratio, Price-to-sales TTM, Total share capital, Circulating shares, Tradable circulating shares, Aggregate market value, Circulation market value
Financial report disclosure date	Finance data	Stock ID, Latest disclosure date, Reporting period, Scheduled disclosure date, Actual disclosure date, Disclosure modification date
Top 10 shareholders data	Other reference data	Stock ID, Announcement date, End date, Shareholder name, Holding amount, Holding ratio
Top 10 floating shareholders data	Other reference data	Stock ID, Announcement date, End date, Shareholder name, Holding amount
Daily top trading list by institution	Other reference data	Stock ID, Trading date, Institution name, Trading amount—buy, Trade ratio—buy, Trading amount—sell, Trade ratio—sell, Net turnover
Daily top transaction detail	Other reference data	Stock ID, Trading date, Stock name, Closing price, Price change percentage, Turnover rate, Amount—overall, On-list amount—sell, On-list amount—buy, On-list turnover, On-list net trading amount, On-list net trading ratio, On-list net turnover ratio, Circulation market value, Reason
Block trade transaction data	Other reference data	Stock ID, Trading date, Price, Volume, Amount, Buyer, Seller
Public fund positioning data	Other reference data	Fund ID, Announcement date, End date, Stock ID, Market value, Volume, Market value ratio, Circulation market value ratio
Basic information of public fund management companies	Other reference data	Company name, Short name, Province, City, Address, Phone, Office
Basic information of public fund positioning	Other reference data	Fund ID, Name, Management company, Custodian, Fund type, Founded date, Due date, List date, Issued date, Delist date, Issued amount, Fee, Duration, Value, Min Amount, Expecting return, Benchmark

The Table 1 lists the field information of each data table as well as which category the data table belongs to.

## Methods

In this section, we present the proposed methods and the design of the proposed solution. Moreover, we also introduce the architecture design as well as algorithmic and implementation details.

### Problem statement

We analyzed the best possible approach for predicting short-term price trends from different aspects: feature engineering, financial domain knowledge, and prediction algorithm. Then we addressed three research questions in each aspect, respectively: How can feature engineering benefit model prediction accuracy? How do findings from the financial domain benefit prediction model design? And what is the best algorithm for predicting short-term price trends?

The first research question is about feature engineering. We would like to know how the feature selection method benefits the performance of prediction models. From the abundance of the previous works, we can conclude that stock price data embedded with a high level of noise, and there are also correlations between features, which makes the price prediction notoriously difficult. That is also the primary reason for most of the previous works introduced the feature engineering part as an optimization module.

The second research question is evaluating the effectiveness of findings we extracted from the financial domain. Different from the previous works, besides the common evaluation of data models such as the training costs and scores, our evaluation will emphasize the effectiveness of newly added features that we extracted from the financial domain. We introduce some features from the financial domain. While we only obtained some specific findings from previous works, and the related raw data needs to be processed into usable features. After extracting related features from the financial domain, we combine the features with other common technical indices for voting out the features with a higher impact. There are numerous features said to be effective from the financial domain, and it would be impossible for us to cover all of them. Thus, how to appropriately convert the findings from the financial domain to a data processing module of our system design is a hidden research question that we attempt to answer.

The third research question is that which algorithms are we going to model our data? From the previous works, researchers have been putting efforts into the exact price prediction. We decompose the problem into predicting the trend and then the exact number. This paper focuses on the first step. Hence, the objective has been converted to resolve a binary classification problem, meanwhile, finding an effective way to eliminate the negative effect brought by the high level of noise. Our approach is to decompose the complex problem into sub-problems which have fewer dependencies and resolve them one by one, and then compile the resolutions into an ensemble model as an aiding system for investing behavior reference.

In the previous works, researchers have been using a variety of models for predicting stock price trends. While most of the best-performed models are based on machine learning techniques, in this work, we will compare our approach with the outperformed machine learning models in the evaluation part and find the solution for this research question.

### Proposed solution

**Fig. 2:**
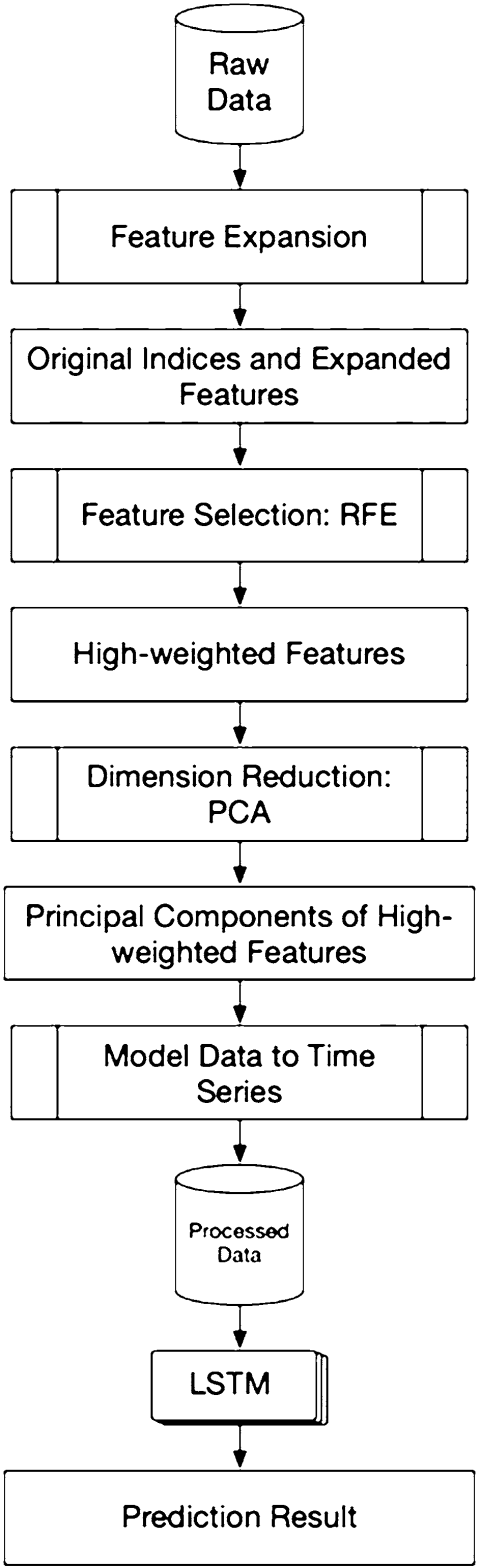
High-level architecture of the proposed solution

The high-level architecture of our proposed solution could be separated into three parts. First is the feature selection part, to guarantee the selected features are highly effective. Second, we look into the data and perform the dimensionality reduction. And the last part, which is the main contribution of our work is to build a prediction model of target stocks. Figure 2 depicts a high-level architecture of the proposed solution.

There are ways to classify different categories of stocks. Some investors prefer long-term investments, while others show more interest in short-term investments. It is common to see the stock-related reports showing an average performance, while the stock price is increasing drastically; this is one of the phenomena that indicate the stock price prediction has no fixed rules, thus finding effective features before training a model on data is necessary.

In this research, we focus on the short-term price trend prediction. Currently, we only have the raw data with no labels. So, the very first step is to label the data. We mark the price trend by comparing the current closing price with the closing price of n trading days ago, the range of n is from 1 to 10 since our research is focusing on the short-term. If the price trend goes up, we mark it as 1 or mark as 0 in the opposite case. To be more specified, we use the indices from the indices of *n *− *1*_*th*_ day to predict the price trend of the *n*_*th*_ day.

According to the previous works, some researchers who applied both financial domain knowledge and technical methods on stock data were using rules to filter the high-quality stocks. We referred to their works and exploited their rules to contribute to our feature extension design.

However, to ensure the best performance of the prediction model, we will look into the data first. There are a large number of features in the raw data; if we involve all the features into our consideration, it will not only drastically increase the computational complexity but will also cause side effects if we would like to perform unsupervised learning in further research. So, we leverage the recursive feature elimination (RFE) to ensure all the selected features are effective.

We found most of the previous works in the technical domain were analyzing all the stocks, while in the financial domain, researchers prefer to analyze the specific scenario of investment, to fill the gap between the two domains, we decide to apply a feature extension based on the findings we gathered from the financial domain before we start the RFE procedure.

Since we plan to model the data into time series, the number of the features, the more complex the training procedure will be. So, we will leverage the dimensionality reduction by using randomized PCA at the beginning of our proposed solution architecture.

### Detailed technical design elaboration

**Fig. 3:**
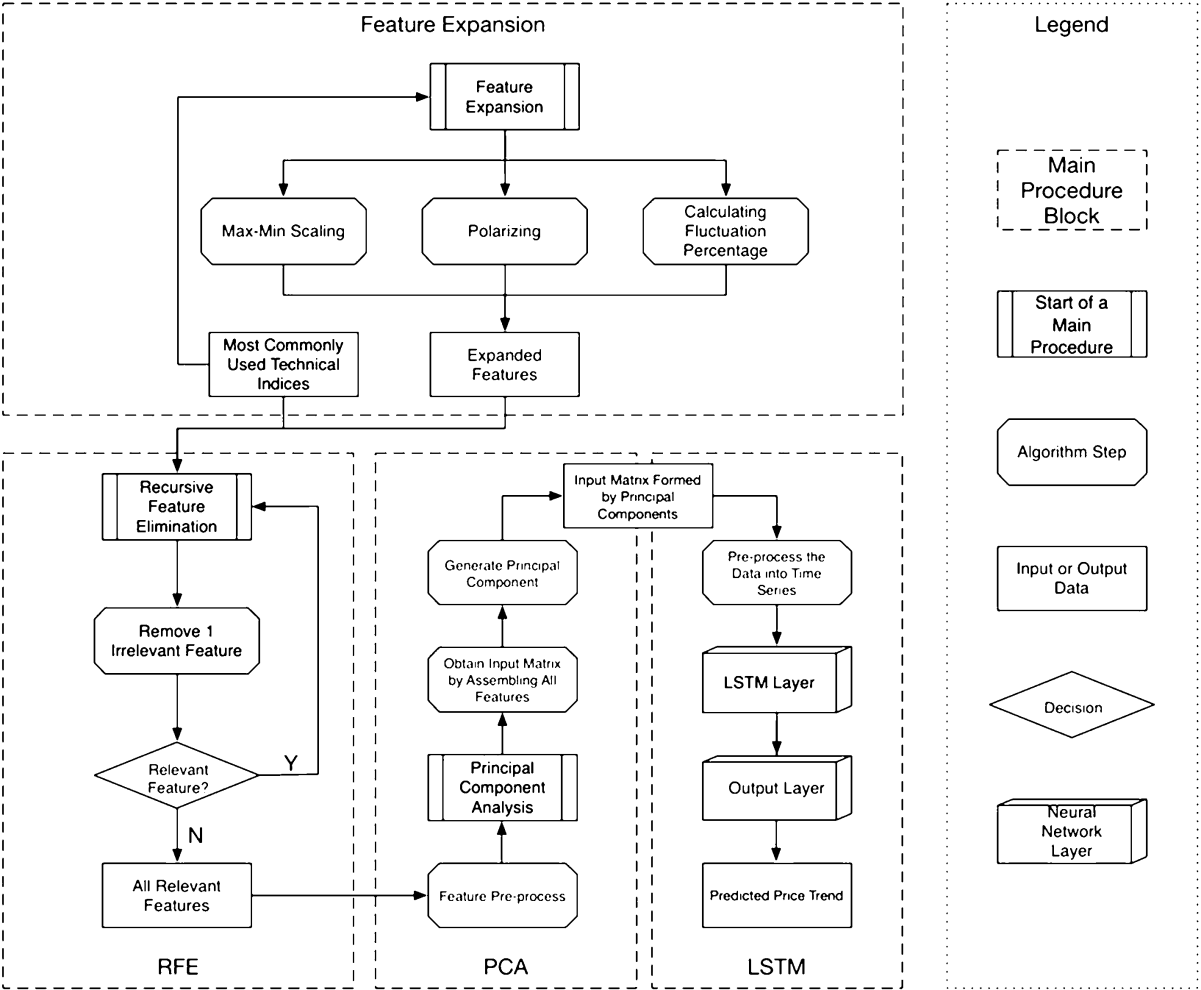
Detailed technical design of the proposed solution

This section provides an elaboration of the detailed technical design as being a comprehensive solution based on utilizing, combining, and customizing several existing data preprocessing, feature engineering, and deep learning techniques. Figure 3 provides the detailed technical design from data processing to prediction, including the data exploration. We split the content by main procedures, and each procedure contains algorithmic steps. Algorithmic details are elaborated in the next section. The contents of this section will focus on illustrating the data workflow.

Based on the literature review, we select the most commonly used technical indices and then feed them into the feature extension procedure to get the expanded feature set. We will select the most effective *i* features from the expanded feature set. Then we will feed the data with *i* selected features into the PCA algorithm to reduce the dimension into *j* features. After we get the best combination of *i* and *j*, we process the data into finalized the feature set and feed them into the LSTM [10] model to get the price trend prediction result.

The novelty of our proposed solution is that we will not only apply the technical method on raw data but also carry out the feature extensions that are used among stock market investors. Details on feature extension are given in the next subsection. Experiences gained from applying and optimizing deep learning based solutions in [37, 38] were taken into account while designing and customizing feature engineering and deep learning solution in this work.

#### Applying feature extension

The first main procedure in Fig. 3 is the feature extension. In this block, the input data is the most commonly used technical indices concluded from related works. The three feature extension methods are max–min scaling, polarizing, and calculating fluctuation percentage. Not all the technical indices are applicable for all three of the feature extension methods; this procedure only applies the meaningful extension methods on technical indices. We choose meaningful extension methods while looking at how the indices are calculated. The technical indices and the corresponding feature extension methods are illustrated in Table 2.

**Table 2:** Feature extension method selection

Feature	Polarize	Max–min scale	Fluctuation percentage
Price change			
Price change percentage			
Volume		√	
Amount		√	
SMA 10		√	√
MACD	√		
MACD SIGNAL	√		
MACD HIST	√		
CCI 24	√		
MTM 10	√		√
ROC 10	√		√
RSI 5		√	√
WNR 9	√	√	
SLOWK		√	√
SLOWD		√	√
ADOSC	√	√	
AR 26		√	
BR 26		√	
VR 26		√	√
BIAS 20	√		

After the feature extension procedure, the expanded features will be combined with the most commonly used technical indices, i.e., input data with output data, and feed into RFE block as input data in the next step.

#### Applying recursive feature elimination

After the feature extension above, we explore the most effective *i* features by using the Recursive Feature Elimination (RFE) algorithm [6]. We estimate all the features by two attributes, coefficient, and feature importance. We also limit the features that remove from the pool by one, which means we will remove one feature at each step and retain all the relevant features. Then the output of the RFE block will be the input of the next step, which refers to PCA.

#### Applying principal component analysis (PCA)

The very first step before leveraging PCA is feature pre-processing. Because some of the features after RFE are percentage data, while others are very large numbers, i.e., the output from RFE are in different units. It will affect the principal component extraction result. Thus, before feeding the data into the PCA algorithm [8], a feature pre-processing is necessary. We also illustrate the effectiveness and methods comparison in “Results” section.

After performing feature pre-processing, the next step is to feed the processed data with selected *i* features into the PCA algorithm to reduce the feature matrix scale into *j* features. This step is to retain as many effective features as possible and meanwhile eliminate the computational complexity of training the model. This research work also evaluates the best combination of *i* and *j,* which has relatively better prediction accuracy, meanwhile, cuts the computational consumption. The result can be found in the “Results” section, as well. After the PCA step, the system will get a reshaped matrix with *j* columns.

#### Fitting long short-term memory (LSTM) model

PCA reduced the dimensions of the input data, while the data pre-processing is mandatory before feeding the data into the LSTM layer. The reason for adding the data pre-processing step before the LSTM model is that the input matrix formed by principal components has no time steps. While one of the most important parameters of training an LSTM is the number of time steps. Hence, we have to model the matrix into corresponding time steps for both training and testing dataset.

After performing the data pre-processing part, the last step is to feed the training data into LSTM and evaluate the performance using testing data. As a variant neural network of RNN, even with one LSTM layer, the NN structure is still a deep neural network since it can process sequential data and memorizes its hidden states through time. An LSTM layer is composed of one or more LSTM units, and an LSTM unit consists of cells and gates to perform classification and prediction based on time series data.

The LSTM structure is formed by two layers. The input dimension is determined by j after the PCA algorithm. The first layer is the input LSTM layer, and the second layer is the output layer. The final output will be 0 or 1 indicates if the stock price trend prediction result is going down or going up, as a supporting suggestion for the investors to perform the next investment decision.

#### Design discussion

Feature extension is one of the novelties of our proposed price trend predicting system. In the feature extension procedure, we use technical indices to collaborate with the heuristic processing methods learned from investors, which fills the gap between the financial research area and technical research area.

Since we proposed a system of price trend prediction, feature engineering is extremely important to the final prediction result. Not only the feature extension method is helpful to guarantee we do not miss the potentially correlated feature, but also feature selection method is necessary for pooling the effective features. The more irrelevant features are fed into the model, the more noise would be introduced. Each main procedure is carefully considered contributing to the whole system design.

Besides the feature engineering part, we also leverage LSTM, the state-of-the-art deep learning method for time-series prediction, which guarantees the prediction model can capture both complex hidden pattern and the time-series related pattern.

It is known that the training cost of deep learning models is expansive in both time and hardware aspects; another advantage of our system design is the optimization procedure—PCA. It can retain the principal components of the features while reducing the scale of the feature matrix, thus help the system to save the training cost of processing the large time-series feature matrix.

#### Algorithm elaboration

This section provides comprehensive details on the algorithms we built while utilizing and customizing different existing techniques. Details about the terminologies, parameters, as well as optimizers. From the legend on the right side of Fig. 3, we note the algorithm steps as octagons, all of them can be found in this “Algorithm elaboration” section.

Before dive deep into the algorithm steps, here is the brief introduction of data pre-processing: since we will go through the supervised learning algorithms, we also need to program the ground truth. The ground truth of this research is programmed by comparing the closing price of the current trading date with the closing price of the previous trading date the users want to compare with. Label the price increase as 1, else the ground truth will be labeled as 0. Because this research work is not only focused on predicting the price trend of a specific period of time but short-term in general, the ground truth processing is according to a range of trading days. While the algorithms will not change with the prediction term length, we can regard the term length as a parameter.

The algorithmic detail is elaborated, respectively, the first algorithm is the hybrid feature engineering part for preparing high-quality training and testing data. It corresponds to the Feature extension, RFE, and PCA blocks in Fig. 3. The second algorithm is the LSTM procedure block, including time-series data pre-processing, NN constructing, training, and testing.

#### Algorithm 1: Short-term stock market price trend prediction—applying feature engineering using FE + RFE + PCA

The function FE is corresponding to the feature extension block. For the feature extension procedure, we apply three different processing methods to translate the findings from the financial domain to a technical module in our system design. While not all the indices are applicable for expanding, we only choose the proper method(s) for certain features to perform the feature extension (FE), according to Table 2.

Normalize method preserves the relative frequencies of the terms, and transform the technical indices into the range of [0, 1]. Polarize is a well-known method often used by real-world investors, sometimes they prefer to consider if the technical index value is above or below zero, we program some of the features using polarize method and prepare for RFE. Max-min (or min-max) [35] scaling is a transformation method often used as an alternative to zero mean and unit variance scaling. Another well-known method used is fluctuation percentage, and we transform the technical indices fluctuation percentage into the range of [− 1, 1].

The function RFE () in the first algorithm refers to recursive feature elimination. Before we perform the training data scale reduction, we will have to make sure that the features we selected are effective. Ineffective features will not only drag down the classification precision but also add more computational complexity. For the feature selection part, we choose recursive feature elimination (RFE). As [45] explained, the process of recursive feature elimination can be split into the ranking algorithm, resampling, and external validation.

For the ranking algorithm, it fits the model to the features and ranks by the importance to the model. We set the parameter to retain *i* numbers of features, and at each iteration of feature selection retains *Si* top-ranked features, then refit the model and assess the performance again to begin another iteration. The ranking algorithm will eventually determine the top *Si* features.

The RFE algorithm is known to have suffered from the over-fitting problem. To eliminate the over-fitting issue, we will run the RFE algorithm multiple times on randomly selected stocks as the training set and ensure all the features we select are high-weighted. This procedure is called data resampling. Resampling can be built as an optimization step as an outer layer of the RFE algorithm.

The last part of our hybrid feature engineering algorithm is for optimization purposes. For the training data matrix scale reduction, we apply Randomized principal component analysis (PCA) [31], before we decide the features of the classification model.

Financial ratios of a listed company are used to present the growth ability, earning ability, solvency ability, etc. Each financial ratio consists of a set of technical indices, each time we add a technical index (or feature) will add another column of data into the data matrix and will result in low training efficiency and redundancy. If non-relevant or less relevant features are included in training data, it will also decrease the precision of classification.
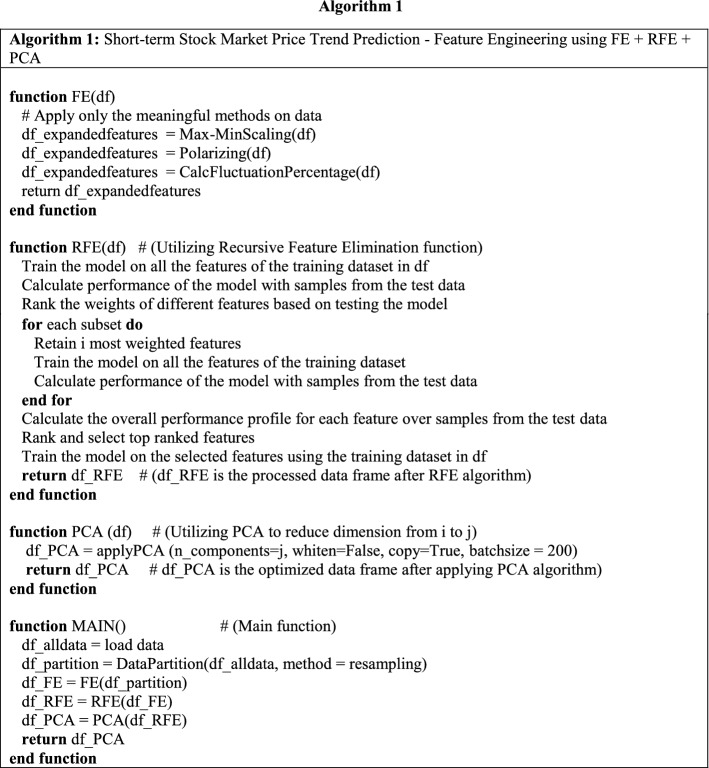

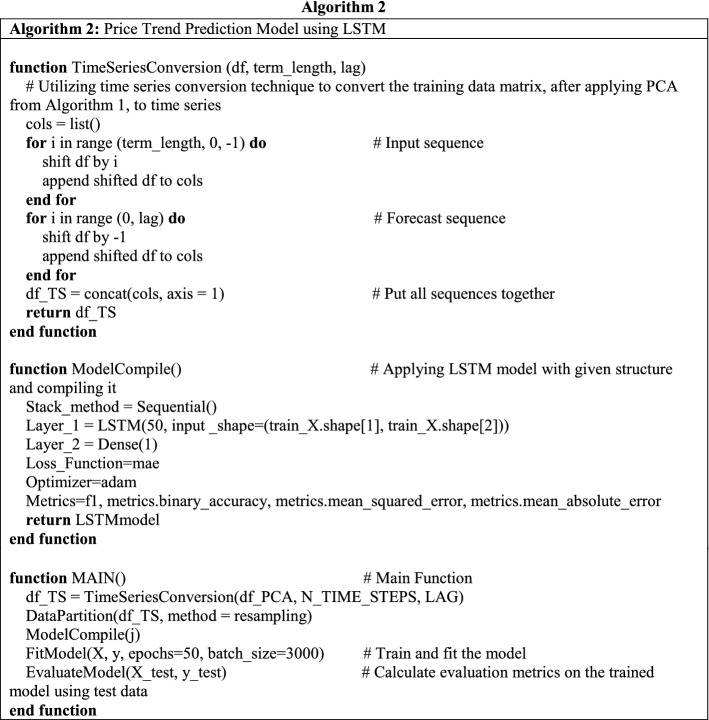


The above equation represents the explanation power of principal components extracted by PCA method for original data. If an ACR is below 85%, the PCA method would be unsuitable due to a loss of original information. Because the covariance matrix is sensitive to the order of magnitudes of data, there should be a data standardize procedure before performing the PCA. The commonly used standardized methods are mean-standardization and normal-standardization and are noted as given below:

- Mean-standardization: $X_{ij}^{*} = X_{ij} /\overline{{X_{j} }}$, which $\overline{{X_{j} }}$ represents the mean value.
- Normal-standardization: $X_{ij}^{*} = (X_{ij} - \overline{{X_{j} }} )/s_{j}$, which $\overline{{X_{j} }}$ represents the mean value, and $s_{j}$ is the standard deviation.

The array *fe_array* is defined according to Table 2, row number maps to the features, columns 0, 1, 2, 3 note for the extension methods of normalize, polarize, max–min scale, and fluctuation percentage, respectively. Then we fill in the values for the array by the rule where 0 stands for no necessity to expand and 1 for features need to apply the corresponding extension methods. The final algorithm of data preprocessing using RFE and PCA can be illustrated as Algorithm 1.

#### Algorithm 2: Price trend prediction model using LSTM

After the principal component extraction, we will get the scale-reduced matrix, which means *i* most effective features are converted into *j* principal components for training the prediction model. We utilized an LSTM model and added a conversion procedure for our stock price dataset. The detailed algorithm design is illustrated in Alg 2. The function *TimeSeriesConversion* () converts the principal components matrix into time series by shifting the input data frame according to the number of time steps [3], i.e., term length in this research. The processed dataset consists of the input sequence and forecast sequence. In this research, the parameter of *LAG* is 1, because the model is detecting the pattern of features fluctuation on a daily basis. Meanwhile, the *N_TIME_STEPS* is varied from 1 trading day to 10 trading days. The functions *DataPartition (), FitModel (), EvaluateModel ()* are regular steps without customization. The NN structure design, optimizer decision, and other parameters are illustrated in function *ModelCompile ()*.

## Results

Some procedures impact the efficiency but do not affect the accuracy or precision and vice versa, while other procedures may affect both efficiency and prediction result. To fully evaluate our algorithm design, we structure the evaluation part by main procedures and evaluate how each procedure affects the algorithm performance. First, we evaluated our solution on a machine with 2.2 GHz i7 processor, with 16 GB of RAM. Furthermore, we also evaluated our solution on Amazon EC2 instance, 3.1 GHz Processor with 16 vCPUs, and 64 GB RAM.

In the implementation part, we expanded 20 features into 54 features, while we retain 30 features that are the most effective. In this section, we discuss the evaluation of feature selection. The dataset was divided into two different subsets, i.e., training and testing datasets. Test procedure included two parts, one testing dataset is for feature selection, and another one is for model testing. We note the feature selection dataset and model testing dataset as DS_test_f and DS_test_m, respectively.

We randomly selected two-thirds of the stock data by stock ID for RFE training and note the dataset as DS_train_f; all the data consist of full technical indices and expanded features throughout 2018. The estimator of the RFE algorithm is SVR with linear kernels. We rank the 54 features by voting and get 30 effective features then process them using the PCA algorithm to perform dimension reduction and reduce the features into 20 principal components. The rest of the stock data forms the testing dataset DS_test_f to validate the effectiveness of principal components we extracted from selected features. We reformed all the data from 2018 as the training dataset of the data model and noted as DS_train_m. The model testing dataset DS_test_m consists of the first 3 months of data in 2019, which has no overlap with the dataset we utilized in the previous steps. This approach is to prevent the hidden problem caused by overfitting.

### Term length

To build an efficient prediction model, instead of the approach of modeling the data to time series, we determined to use 1 day ahead indices data to predict the price trend of the next day. We tested the RFE algorithm on a range of short-term from 1 day to 2 weeks (ten trading days), to evaluate how the commonly used technical indices correlated to price trends. For evaluating the prediction term length, we fully expanded the features as Table 2, and feed them to RFE. During the test, we found that different length of the term has a different level of sensitive-ness to the same indices set.

**Fig. 4:**
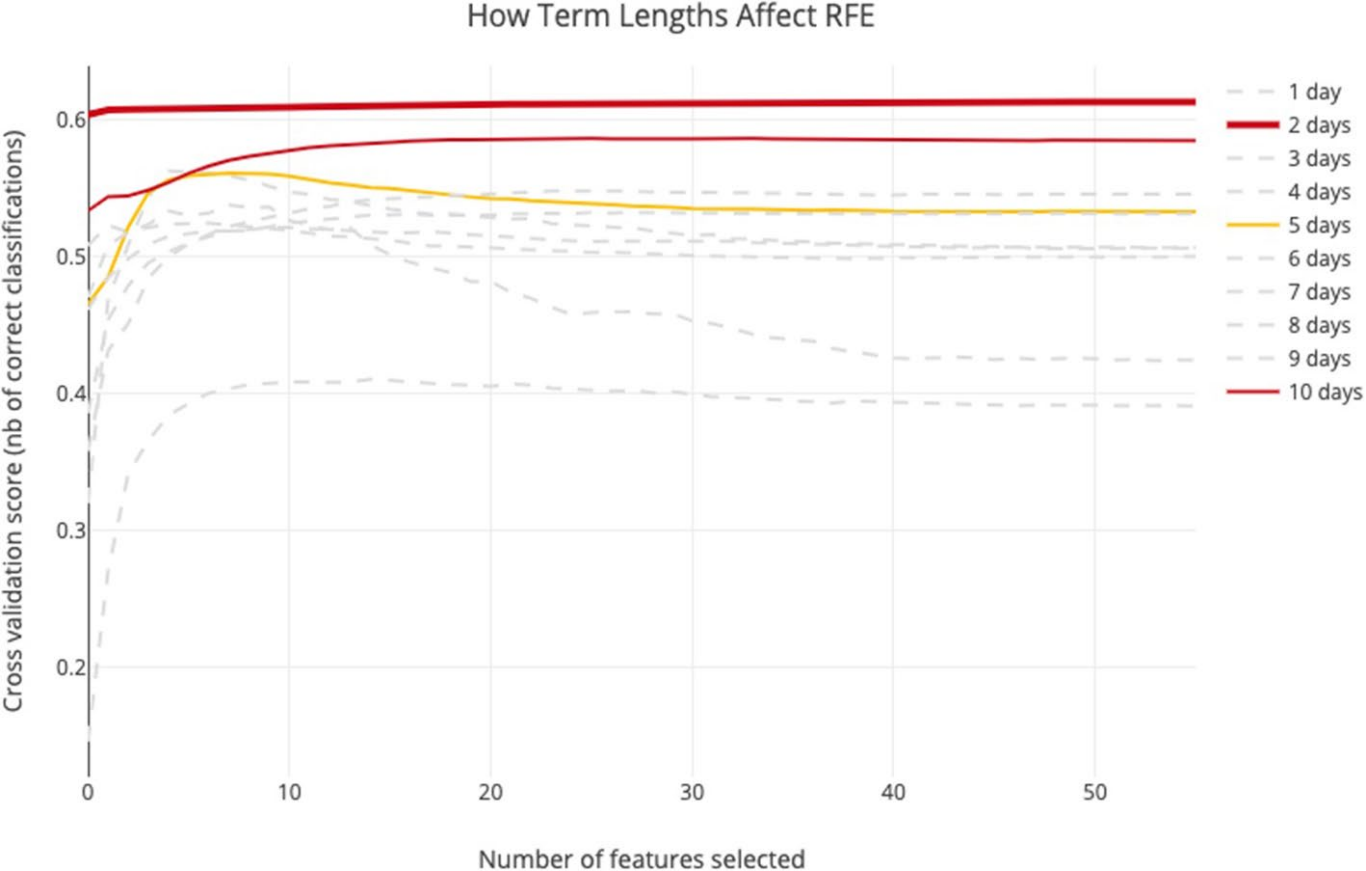
How do term lengths affect the cross-validation score of RFE

We get the close price of the first trading date and compare it with the close price of the *n*_*th* trading date. Since we are predicting the price trend, we do not consider the term lengths if the cross-validation score is below 0.5. And after the test, as we can see from Fig. 4, there are three-term lengths that are most sensitive to the indices we selected from the related works. They are *n *= {2, 5, 10}, which indicates that price trend prediction of every other day, 1 week, and 2 weeks using the indices set are likely to be more reliable.

While these curves have different patterns, for the length of 2 weeks, the cross-validation score increases with the number of features selected. If the prediction term length is 1 week, the cross-validation score will decrease if selected over 8 features. For every other day price trend prediction, the best cross-validation score is achieved by selecting 48 features. Biweekly prediction requires 29 features to achieve the best score. In Table 3, we listed the top 15 effective features for these three-period lengths. If we predict the price trend of every other day, the cross-validation score merely fluctuates with the number of features selected. So, in the next step, we will evaluate the RFE result for these three-term lengths, as shown in Fig. 4.

**Table 3:** Effective features corresponding to term lengths

Relevant ranking	Every other day	Weekly	Bi-weekly
1st	Up_down	SLOWK_maxmin	MTM_10_plr
2nd	Change	SLOWK	ROC_10_plr
3rd	pct_chg	SLOWD_maxmin	WNR_9
4th	Low	RSI_5_maxmin	WNR_9_maxmin
5th	RSI_5_flc	SLOWD	SLOWK
6th	Open	RSI_5	SLOWK_maxmin
7th	Amount	SLOWK_flc	ROC_10
8th	Amount_maxmin	WNR_9_maxmin	SLOWD_flc
9th	Vol	WNR_9	WNR_9_flc
10th	BIAS_20_maxmin	CCI_24	RSI_5
11th	High	BIAS_20_maxmin	BIAS_20_maxmin
12th	Vol_maxmin	BIAS_20	RSI_5_maxmin
13th	ROC_10	ADOSC_maxmin	BIAS_20
14th	ADOSC_maxmin	ADOSC	SMA_10
15th	ADOSC	WNR_9_flc	SLOWD
…	…		…
Number of Features Selected	48 features selected	8 features selected	29 features selected

We compare the output feature set of RFE with the all-original feature set as a baseline, the all-original feature set consists of n features and we choose *n* most effective features from RFE output features to evaluate the result using linear SVR. We used two different approaches to evaluate feature effectiveness. The first method is to combine all the data into one large matrix and evaluate them by running the RFE algorithm once. Another method is to run RFE for each individual stock and calculate the most effective features by voting.

### Feature extension and RFE

From the result of the previous subsection, we can see that when predicting the price trend for every other day or biweekly, the best result is achieved by selecting a large number of features. Within the selected features, some features processed from extension methods have better ranks than original features, which proves that the feature extension method is useful for optimizing the model. The feature extension affects both precision and efficiency, while in this part, we only discuss the precision aspect and leave efficiency part in the next step since PCA is the most effective method for training efficiency optimization in our design. We involved an evaluation of how feature extension affects RFE and use the test result to measure the improvement of involving feature extension.

We further test the effectiveness of feature extension, i.e., if polarize, max–min scale, and calculate fluctuation percentage works better than original technical indices. The best case to leverage this test is the weekly prediction since it has the least effective feature selected. From the result we got from the last section, we know the best cross-validation score appears when selecting 8 features. The test consists of two steps, and the first step is to test the feature set formed by original features only, in this case, only SLOWK, SLOWD, and RSI_5 are included. The next step is to test the feature set of all 8 features we selected in the previous subsection. We leveraged the test by defining the simplest DNN model with three layers.

**Fig. 5:**
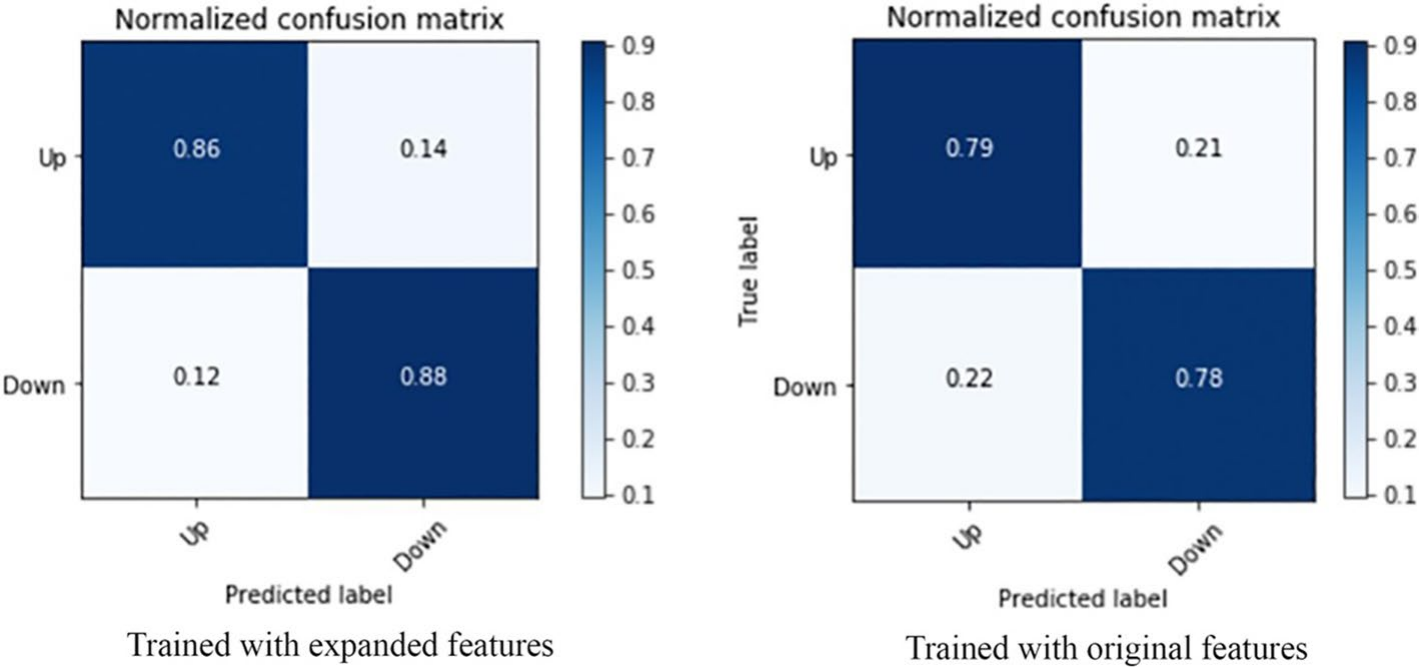
Confusion matrix of validating feature extension effectiveness

The normalized confusion matrix of testing the two feature sets are illustrated in Fig. 5. The left one is the confusion matrix of the feature set with expanded features, and the right one besides is the test result of using original features only. Both precisions of true positive and true negative have been improved by 7% and 10%, respectively, which proves that our feature extension method design is reasonably effective.

### Feature reduction using principal component analysis

PCA will affect the algorithm performance on both prediction accuracy and training efficiency, while this part should be evaluated with the NN model, so we also defined the simplest DNN model with three layers as we used in the previous step to perform the evaluation. This part introduces the evaluation method and result of the optimization part of the model from computational efficiency and accuracy impact perspectives.

**Fig. 6:**
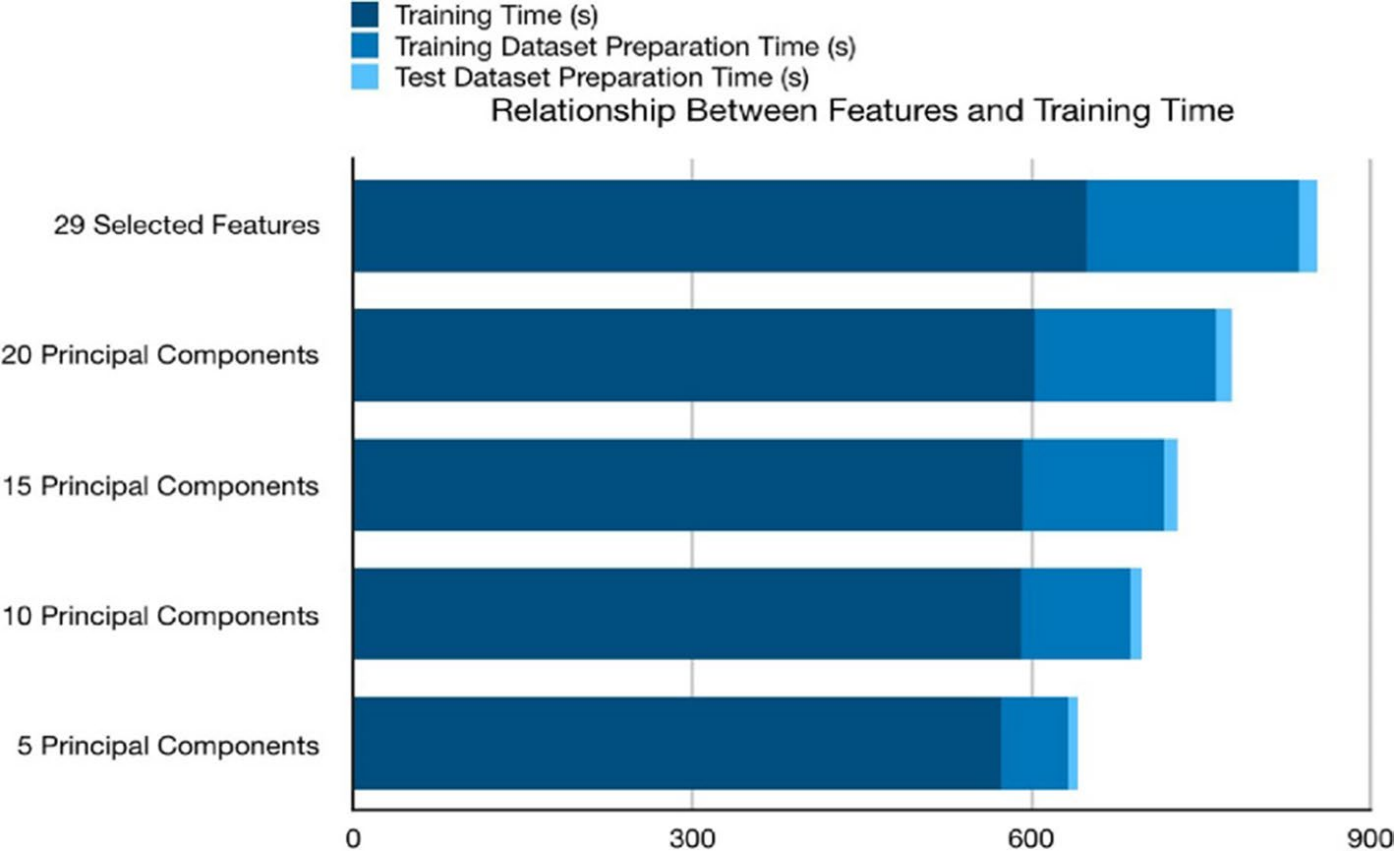
Relationship between feature number and training time

In this section, we will choose bi-weekly prediction to perform a use case analysis, since it has a smoothly increasing cross-validation score curve, moreover, unlike every other day prediction, it has excluded more than 20 ineffective features already. In the first step, we select all 29 effective features and train the NN model without performing PCA. It creates a baseline of the accuracy and training time for comparison. To evaluate the accuracy and efficiency, we keep the number of the principal component as 5, 10, 15, 20, 25. Table 4 recorded how the number of features affects the model training efficiency, then uses the stack bar chart in Fig. 6 to illustrate how PCA affects training efficiency. Table 6 shows accuracy and efficiency analysis on different procedures for the pre-processing of features. The times taken shown in Tables 4, 6 are based on experiments conducted in a standard user machine to show the viability of our solution with limited or average resource availability.

**Table 4:** Relationship between the number of principal components and training efficiency

Number of features	Training dataset preparation time (s)	Test dataset preparation time (s)	Training time (s)	Sum (s)
29 selected features	187.46	16.30	648.53	852.29
20 principal components	160.29	14.24	602.68	777.21
15 principal components	125.20	12.18	591.93	729.31
10 principal components	96.54	10.37	590.76	697.67
5 principal components	59.37	8.22	572.88	640.47

**Fig. 7:**
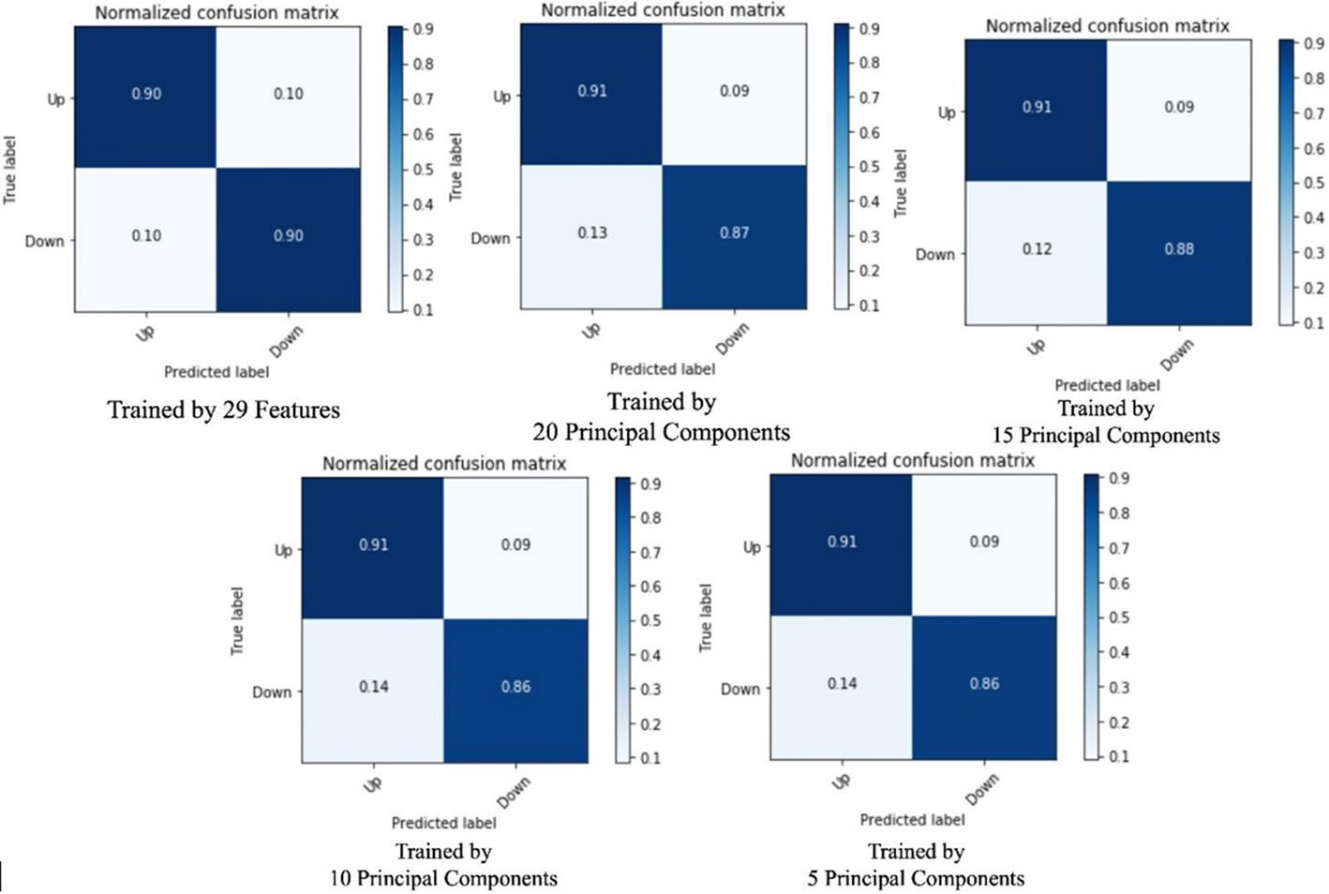
How does the number of principal components affect evaluation results

We also listed the confusion matrix of each test in Fig. 7. The stack bar chart shows that the overall time spends on training the model is decreasing by the number of selected features, while the PCA method is significantly effective in optimizing training dataset preparation. For the time spent on the training stage, PCA is not as effective as the data preparation stage. While there is the possibility that the optimization effect of PCA is not drastic enough because of the simple structure of the NN model.

Table 5 indicates that the overall prediction accuracy is not drastically affected by reducing the dimension. However, the accuracy could not fully support if the PCA has no side effect to model prediction, so we looked into the confusion matrices of test results.

**Table 5:** How does the number of selected features affect the prediction accuracy

Number of selected features	5 principal components	10 principal components	15 principal components	20 principal components	29 selected features
Accuracy	89.03%	89.35%	89.39%	89.30%	90.29%

From Fig. 7 we can conclude that PCA does not have a severe negative impact on prediction precision. The true positive rate and false positive rate are barely be affected, while the false negative and true negative rates are influenced by 2% to 4%. Besides evaluating how the number of selected features affects the training efficiency and model performance, we also leveraged a test upon how data pre-processing procedures affect the training procedure and predicting result. Normalizing and max–min scaling is the most commonly seen data pre-procedure performed before PCA, since the measure units of features are varied, and it is said that it could increase the training efficiency afterward.

**Table 6:** Accuracy and efficiency analysis on feature pre-processing procedures

Feature pre-processing	Overall accuracy (%)	Training dataset preparation time (s)	Testing dataset preparation time (s)	Training time (s)	Sum (s)
Max–min scaling	89.30	160.28	14.24	602.68	777.20
Normalization	78.17	157.63	14.73	596.22	768.58
N/A	78.88	142.17	13.00	595.52	750.69

**Fig. 8:**
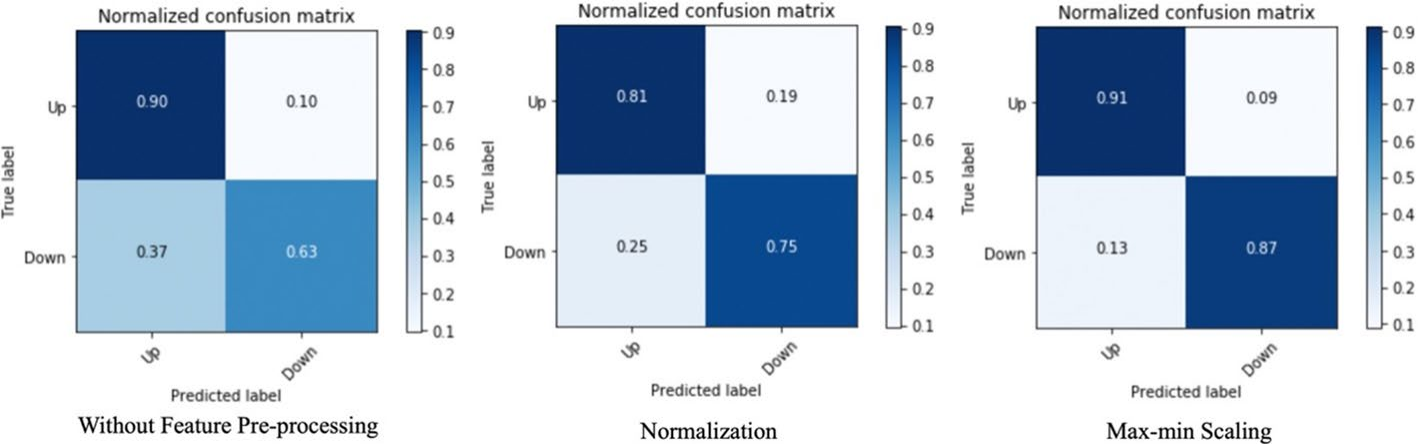
Confusion matrices of different feature pre-processing methods

We leveraged another test on adding pre-procedures before extracting 20 principal components from the original dataset and make the comparison in the aspects of time elapse of training stage and prediction precision. However, the test results lead to different conclusions. In Table 6 we can conclude that feature pre-processing does not have a significant impact on training efficiency, but it does influence the model prediction accuracy. Moreover, the first confusion matrix in Fig. 8 indicates that without any feature pre-processing procedure, the false-negative rate and true negative rate are severely affected, while the true positive rate and false positive rate are not affected. If it performs the normalization before PCA, both true positive rate and true negative rate are decreasing by approximately 10%. This test also proved that the best feature pre-processing method for our feature set is exploiting the max–min scale.

## Discussion

In this section, we discuss and compare the results of our proposed model, other approaches, and the most related works.

### Comparison with related works

From the previous works, we found the most commonly exploited models for short-term stock market price trend prediction are support vector machine (SVM), multilayer perceptron artificial neural network (MLP), Naive Bayes classifier (NB), random forest classifier (RAF) and logistic regression classifier (LR). The test case of comparison is also bi-weekly price trend prediction, to evaluate the best result of all models, we keep all 29 features selected by the RFE algorithm. For MLP evaluation, to test if the number of hidden layers would affect the metric scores, we noted layer number as *n* and tested *n *= {1, 3, 5}, 150 training epochs for all the tests, found slight differences in the model performance, which indicates that the variable of MLP layer number hardly affects the metric scores.

**Fig. 9:**
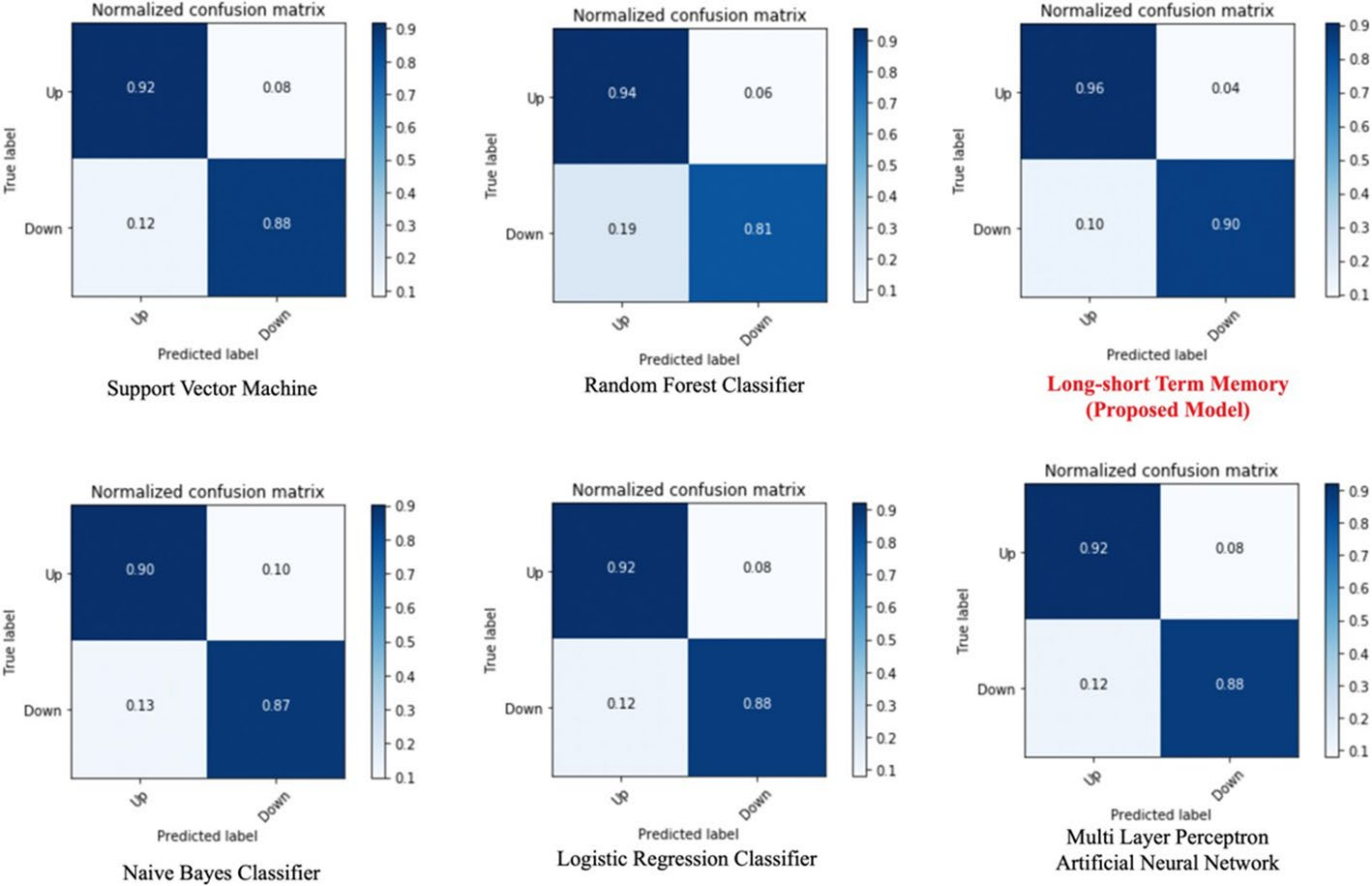
Model prediction comparison—confusion matrices

**Fig. 10:**
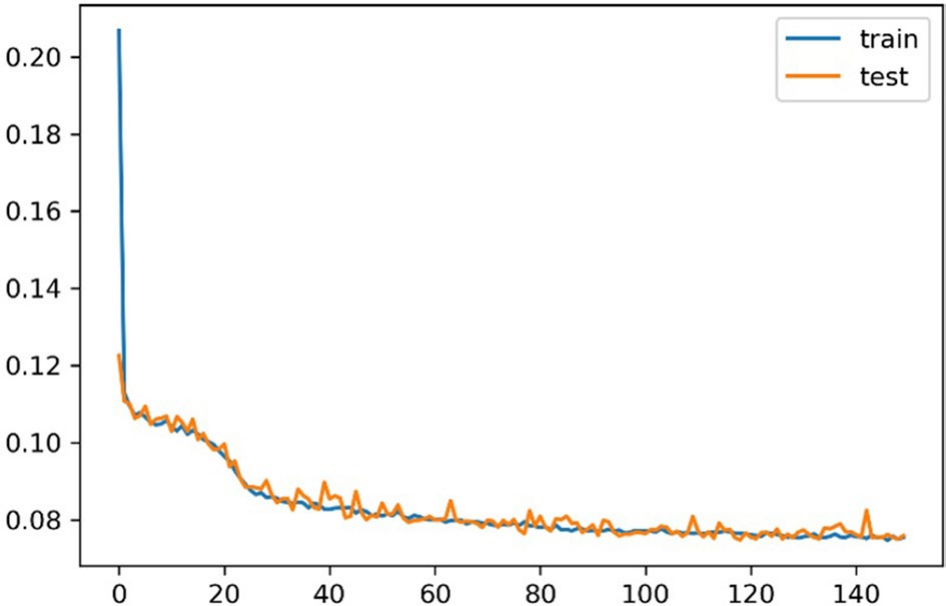
Learning curve of proposed solution

**Fig. 11:**
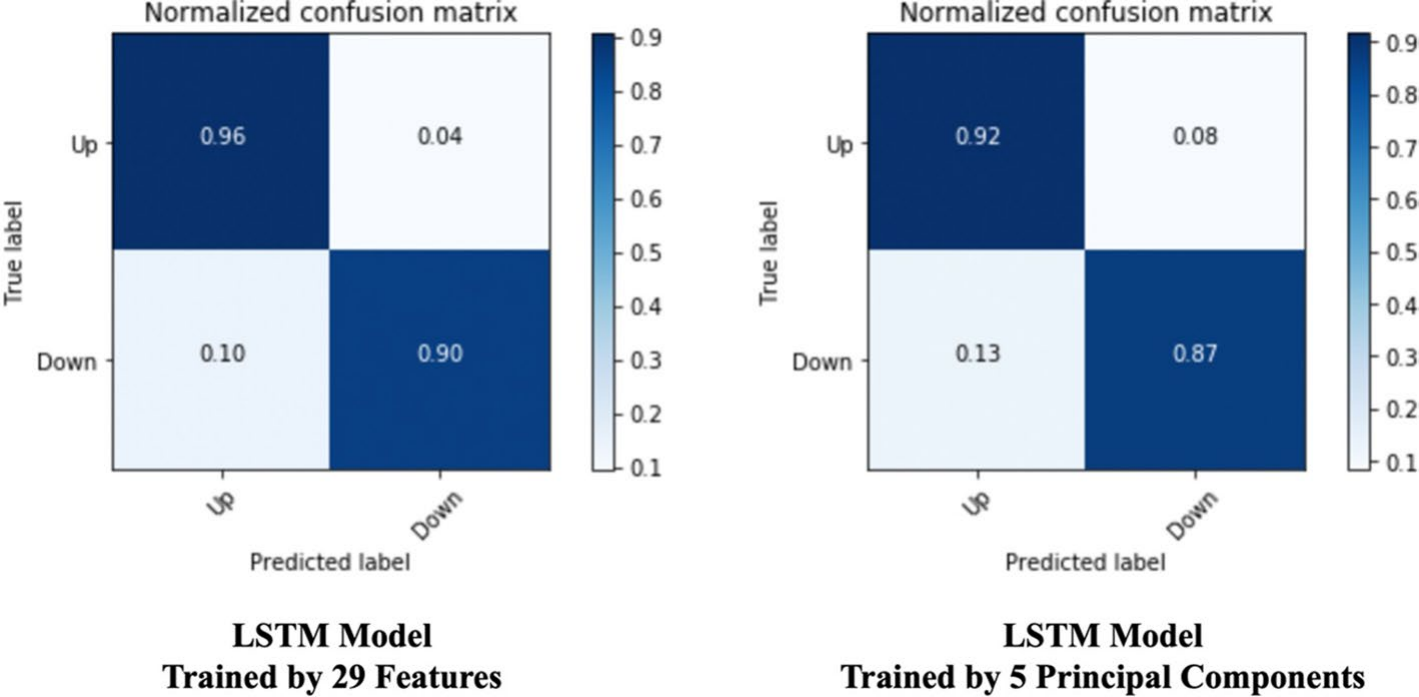
Proposed model prediction precision comparison—confusion matrices

From the confusion matrices in Fig. 9, we can see all the machine learning models perform well when training with the full feature set we selected by RFE. From the perspective of training time, training the NB model got the best efficiency. LR algorithm cost less training time than other algorithms while it can achieve a similar prediction result with other costly models such as SVM and MLP. RAF algorithm achieved a relatively high true-positive rate while the poor performance in predicting negative labels. For our proposed LSTM model, it achieves a binary accuracy of 93.25%, which is a significantly high precision of predicting the bi-weekly price trend. We also pre-processed data through PCA and got five principal components, then trained for 150 epochs. The learning curve of our proposed solution, based on feature engineering and the LSTM model, is illustrated in Fig. 10. The confusion matrix is the figure on the right in Fig. 11, and detailed metrics scores can be found in Table 9.

The detailed evaluate results are recorded in Table 7. We will also initiate a discussion upon the evaluation result in the next section.

**Table 7:** Model performance comparison—metric scores

Model	F1 score	Binary accuracy	TPR (recall)	TNR (specificity)	FPR (fall-out)	FNR (miss rate)
LR	0.90	0.90	0.92	0.88	0.08	0.12
SVM	0.90	0.90	0.92	0.88	0.08	0.12
NB	0.89	0.89	0.90	0.87	0.10	0.13
MLP (Single hidden layer)	0.90	0.90	0.92	0.88	0.08	0.12
MLP (3 hidden layers)	0.90	0.90	0.92	0.87	0.08	0.13
MLP (5 hidden layers)	0.90	0.90	0.92	0.88	0.08	0.12
RAF	0.88	0.88	0.94	0.81	0.06	0.19
Proposed model	0.93	0.93	0.96	0.90	0.04	0.10

Because the resulting structure of our proposed solution is different from most of the related works, it would be difficult to make naïve comparison with previous works. For example, it is hard to find the exact accuracy number of price trend prediction in most of the related works since the authors prefer to show the gain rate of simulated investment. Gain rate is a processed number based on simulated investment tests, sometimes one correct investment decision with a large trading volume can achieve a high gain rate regardless of the price trend prediction accuracy. Besides, it is also a unique and heuristic innovation in our proposed solution, we transform the problem of predicting an exact price straight forward to two sequential problems, i.e., predicting the price trend first, focus on building an accurate binary classification model, construct a solid foundation for predicting the exact price change in future works. Besides the different result structure, the datasets that previous works researched on are also different from our work. Some of the previous works involve news data to perform sentiment analysis and exploit the SE part as another system component to support their prediction model.

The latest related work that can compare is Zubair et al. [47], the authors take multiple r-square for model accuracy measurement. Multiple r-square is also called the coefficient of determination, and it shows the strength of predictor variables explaining the variation in stock return [28]. They used three datasets (KSE 100 Index, Lucky Cement Stock, Engro Fertilizer Limited) to evaluate the proposed multiple regression model and achieved 95%, 89%, and 97%, respectively. Except for the KSE 100 Index, the dataset choice in this related work is individual stocks; thus, we choose the evaluation result of the first dataset of their proposed model.

We listed the leading stock price trend prediction model performance in Table 8, from the comparable metrics, the metric scores of our proposed solution are generally better than other related works. Instead of concluding arbitrarily that our proposed model outperformed other models in related works, we first look into the dataset column of Table 8. By looking into the dataset used by each work [18], only trained and tested their proposed solution on three individual stocks, which is difficult to prove the generalization of their proposed model. Ayo [2] leveraged analysis on the stock data from the New York Stock Exchange (NYSE), while the weakness is they only performed analysis on closing price, which is a feature embedded with high noise. Zubair et al. [47] trained their proposed model on both individual stocks and index price, but as we have mentioned in the previous section, index price only consists of the limited number of features and stock IDs, which will further affect the model training quality. For our proposed solution, we collected sufficient data from the Chinese stock market, and applied FE + RFE algorithm on the original indices to get more effective features, the comprehensive evaluation result of 3558 stock IDs can reasonably explain the generalization and effectiveness of our proposed solution in Chinese stock market. However, the authors of Khaidem and Dey [18] and Ayo [2] chose to analyze the stock market in the United States, Zubair et al. [47] performed analysis on Pakistani stock market price, and we obtained the dataset from Chinese stock market, the policies of different countries might impact the model performance, which needs further research to validate.

**Table 8:** Comparison of proposed solution with related works

Related work	Dataset	Model	Accuracy	Precision	Recall
Khaidem and Dey [18]	Stock price data of AAPL, GE and Samsung Electronics Co. Ltd.	Random forest	0.83	0.82	0.81
Ayo [2]	Close price of stock data from New York Stock Exchange (NYSE)	ARIMA	0.90	0.91	0.92
Zubair et al. [47]	KSE 100 Index Lucky Cement Stock Engro Fertilizer Limited	Multiple regression	0.94	0.95	0.93
*(Proposed solution)*	*Price data of 3558 stock ID from 2017 to 2018 collected from Chinese stock market*	*Proposed Model*—*FE *+ *RFE *+ *PCA *+ *LSTM*	0.93	0.96	0.96

### Proposed model evaluation—PCA effectiveness

Besides comparing the performance across popular machine learning models, we also evaluated how the PCA algorithm optimizes the training procedure of the proposed LSTM model. We recorded the confusion matrices comparison between training the model by 29 features and by five principal components in Fig. 11. The model training using the full 29 features takes 28.5 s per epoch on average. While it only takes 18 s on average per epoch training on the feature set of five principal components. PCA has significantly improved the training efficiency of the LSTM model by 36.8%. The detailed metrics data are listed in Table 9. We will leverage a discussion in the next section about complexity analysis.

**Table 9:** Proposed model performance comparison—with and without PCA

Metrics name	LSTM trained on 29 features	LSTM trained on 5 principal components
Loss	0.0702	0.0848
F1 score	0.9323	0.9194
Binary accuracy	0.9325	0.9193
MSE	0.0669	0.0772
MAE	0.0702	0.0848
TPR	0.96	0.92
TNR	0.90	0.91
FPR	0.04	0.08
FNR	0.10	0.09

### Complexity analysis of proposed solution

This section analyzes the complexity of our proposed solution. The Long Short-term Memory is different from other NNs, and it is a variant of standard RNN, which also has time steps with memory and gate architecture. In the previous work [46], the author performed an analysis of the RNN architecture complexity. They introduced a method to regard RNN as a directed acyclic graph and proposed a concept of recurrent depth, which helps perform the analysis on the intricacy of RNN.

The recurrent depth is a positive rational number, and we denote it as $$d_{rc}$$. As the growth of $$n$$$$d_{rc}$$ measures, the nonlinear transformation average maximum number of each time step. We then unfold the directed acyclic graph of RNN and denote the processed graph as $$g_{c}$$, meanwhile, denote $$C(g_{c} )$$ as the set of directed cycles in this graph. For the vertex $$v$$, we note $$\sigma_{s} (v)$$ as the sum of edge weights and $$l(v)$$ as the length. The equation below is proved under a mild assumption, which could be found in [46].

$$d_{rc} = \max_{{v \in C(g_{c} )}} \frac{l(v)}{{\sigma_{s} (v)}}$$

They also found that another crucial factor that impacts the performance of LSTM, which is the recurrent skip coefficients. We note $s_{rc}$ as the reciprocal of the recurrent skip coefficient. Please be aware that $s_{rc}$ is also a positive rational number.

$$s_{rc} = \min_{{v \in C(g_{c} )}} \frac{{\sigma_{s} (v)}}{l(v)}$$

According to the above definition, our proposed model is a 2-layers stacked LSTM, which $d_{rc} = 2$ and $s_{rc} = 1$. From the experiments performed in previous work, the authors also found that when facing the problems of long-term dependency, LSTMs may benefit from decreasing the reciprocal of recurrent skip coefficients and from increasing recurrent depth. The empirical findings above mentioned are useful to enhance the performance of our proposed model further.

## Conclusion

This work consists of three parts: data extraction and pre-processing of the Chinese stock market dataset, carrying out feature engineering, and stock price trend prediction model based on the long short-term memory (LSTM). We collected, cleaned-up, and structured 2 years of Chinese stock market data. We reviewed different techniques often used by real-world investors, developed a new algorithm component, and named it as feature extension, which is proved to be effective. We applied the feature expansion (FE) approaches with recursive feature elimination (RFE), followed by principal component analysis (PCA), to build a feature engineering procedure that is both effective and efficient. The system is customized by assembling the feature engineering procedure with an LSTM prediction model, achieved high prediction accuracy that outperforms the leading models in most related works. We also carried out a comprehensive evaluation of this work. By comparing the most frequently used machine learning models with our proposed LSTM model under the feature engineering part of our proposed system, we conclude many heuristic findings that could be future research questions in both technical and financial research domains.

Our proposed solution is a unique customization as compared to the previous works because rather than just proposing yet another state-of-the-art LSTM model, we proposed a fine-tuned and customized deep learning prediction system along with utilization of comprehensive feature engineering and combined it with LSTM to perform prediction. By researching into the observations from previous works, we fill in the gaps between investors and researchers by proposing a feature extension algorithm before recursive feature elimination and get a noticeable improvement in the model performance.

Though we have achieved a decent outcome from our proposed solution, this research has more potential towards research in future. During the evaluation procedure, we also found that the RFE algorithm is not sensitive to the term lengths other than 2-day, weekly, biweekly. Getting more in-depth research into what technical indices would influence the irregular term lengths would be a possible future research direction. Moreover, by combining latest sentiment analysis techniques with feature engineering and deep learning model, there is also a high potential to develop a more comprehensive prediction system which is trained by diverse types of information such as tweets, news, and other text-based data.

## Abbreviations

LSTM: Long short term memory
PCA: Principal component analysis
RNN: Recurrent neural networks
ANN: Artificial neural network
DNN: Deep neural network
DTW: Dynamic Time Warping
RFE: Recursive feature elimination
SVM: Support vector machine
CNN: Convolutional neural network
SGD: Stochastic gradient descent
ReLU: Rectified linear unit
MLP: Multi layer perceptron

## References

[1] Atsalakis GS, Valavanis KP (2009). Forecasting stock market short-term trends using a neuro-fuzzy based methodology. Expert Syst Appl.

[2] Ayo CK. Stock price prediction using the ARIMA model. In: 2014 UKSim-AMSS 16th international conference on computer modelling and simulation. 2014. 10.1109/UKSim.2014.67.

[3] Brownlee J. Deep learning for time series forecasting: predict the future with MLPs, CNNs and LSTMs in Python. Machine Learning Mastery. 2018. https://machinelearningmastery.com/time-series-prediction-lstm-recurrent-neural-networks-python-keras/

[4] Eapen J, Bein D, Verma A. Novel deep learning model with CNN and bi-directional LSTM for improved stock market index prediction. In: 2019 IEEE 9th annual computing and communication workshop and conference (CCWC). 2019. pp. 264–70. 10.1109/CCWC.2019.8666592.

[5] Fischer T, Krauss C (2018). Deep learning with long short-term memory networks for financial market predictions. Eur J Oper Res.

[6] Guyon I, Weston J, Barnhill S, Vapnik V (2002). Gene selection for cancer classification using support vector machines. Mach Learn.

[7] Hafezi R, Shahrabi J, Hadavandi E (2015). A bat-neural network multi-agent system (BNNMAS) for stock price prediction: case study of DAX stock price. Appl Soft Comput J.

[8] Halko N, Martinsson PG, Tropp JA (2001). Finding structure with randomness: probabilistic algorithms for constructing approximate matrix decompositions. SIAM Rev.

[9] Hassan MR, Nath B. Stock market forecasting using Hidden Markov Model: a new approach. In: Proceedings—5th international conference on intelligent systems design and applications 2005, ISDA’05. 2005. pp. 192–6. 10.1109/ISDA.2005.85.

[10] Hochreiter S, Schmidhuber J (1997). Long short-term memory. J Neural Comput.

[11] Hsu CM (2013). A hybrid procedure with feature selection for resolving stock/futures price forecasting problems. Neural Comput Appl.

[12] Huang CF, Chang BR, Cheng DW, Chang CH (2012). Feature selection and parameter optimization of a fuzzy-based stock selection model using genetic algorithms. Int J Fuzzy Syst.

[13] Huang CL, Tsai CY (2009). A hybrid SOFM-SVR with a filter-based feature selection for stock market forecasting. Expert Syst Appl.

[14] Idrees SM, Alam MA, Agarwal P (2019). A prediction approach for stock market volatility based on time series data. IEEE Access.

[15] Ince H, Trafalis TB (2008). Short term forecasting with support vector machines and application to stock price prediction. Int J Gen Syst.

[16] Jeon S, Hong B, Chang V (2018). Pattern graph tracking-based stock price prediction using big data. Future Gener Comput Syst.

[17] Kara Y, Acar Boyacioglu M, Baykan ÖK (2011). Predicting direction of stock price index movement using artificial neural networks and support vector machines: the sample of the Istanbul Stock Exchange. Expert Syst Appl.

[18] Khaidem L, Dey SR. Predicting the direction of stock market prices using random forest. 2016. pp. 1–20.

[19] Kim K, Han I (2000). Genetic algorithms approach to feature discretization in artificial neural networks for the prediction of stock price index. Expert Syst Appl.

[20] Lee MC (2009). Using support vector machine with a hybrid feature selection method to the stock trend prediction. Expert Syst Appl.

[21] Lei L (2018). Wavelet neural network prediction method of stock price trend based on rough set attribute reduction. Appl Soft Comput J.

[22] Lin X, Yang Z, Song Y (2009). Expert systems with applications short-term stock price prediction based on echo state networks. Expert Syst Appl.

[23] Liu G, Wang X (2019). A new metric for individual stock trend prediction. Eng Appl Artif Intell.

[24] Liu S, Zhang C, Ma J. CNN-LSTM neural network model for quantitative strategy analysis in stock markets. 2017;1:198–206. 10.1007/978-3-319-70096-0.

[25] Long W, Lu Z, Cui L (2018). Deep learning-based feature engineering for stock price movement prediction. Knowl Based Syst.

[26] Malkiel BG, Fama EF (1970). Efficient capital markets: a review of theory and empirical work. J Finance.

[27] McNally S, Roche J, Caton S. Predicting the price of bitcoin using machine learning. In: Proceedings—26th Euromicro international conference on parallel, distributed, and network-based processing, PDP 2018. pp. 339–43. 10.1109/PDP2018.2018.00060. 10.1109/PDP2018.2018.00114 PMC6116909 30175326

[28] Nagar A, Hahsler M. News sentiment analysis using R to predict stock market trends. 2012. http://past.rinfinance.com/agenda/2012/talk/Nagar+Hahsler.pdf. Accessed 20 July 2019.

[29] Nekoeiqachkanloo H, Ghojogh B, Pasand AS, Crowley M. Artificial counselor system for stock investment. 2019. ArXiv Preprint arXiv:1903.00955.

[30] Ni LP, Ni ZW, Gao YZ (2011). Stock trend prediction based on fractal feature selection and support vector machine. Expert Syst Appl.

[31] Pang X, Zhou Y, Wang P, Lin W, Chang V (2018). An innovative neural network approach for stock market prediction. J Supercomput.

[32] Pimenta A, Nametala CAL, Guimarães FG, Carrano EG (2018). An automated investing method for stock market based on multiobjective genetic programming. Comput Econ.

[33] Piramuthu S (2004). Evaluating feature selection methods for learning in data mining applications. Eur J Oper Res.

[34] Qiu M, Song Y (2016). Predicting the direction of stock market index movement using an optimized artificial neural network model. PLoS ONE.

[35] Scikit-learn. Scikit-learn Min-Max Scaler. 2019. https://scikit-learn.org/stable/modules/generated/sklearn.preprocessing.MinMaxScaler.html. Retrieved 26 July 2020.

[36] Shen J. Thesis, “Short-term stock market price trend prediction using a customized deep learning system”, supervised by M. Omair Shafiq, Carleton University. 2019. 10.1186/s40537-020-00333-6 PMC7467129 32923309

[37] Shen J, Shafiq MO (2018). Deep learning convolutional neural networks with dropout—a parallel approach. ICMLA.

[38] Shen J, Shafiq MO (2019). Learning mobile application usage—a deep learning approach. ICMLA.

[39] Shih D. A study of early warning system in volume burst risk assessment of stock with Big Data platform. In: 2019 IEEE 4th international conference on cloud computing and big data analysis (ICCCBDA). 2019. pp. 244–8.

[40] Sirignano J, Cont R (2018). Universal features of price formation in financial markets: perspectives from deep learning. Ssrn.

[41] Thakur M, Kumar D (2018). A hybrid financial trading support system using multi-category classifiers and random forest. Appl Soft Comput J.

[42] Tsai CF, Hsiao YC (2010). Combining multiple feature selection methods for stock prediction: union, intersection, and multi-intersection approaches. Decis Support Syst.

[43] Tushare API. 2018. https://github.com/waditu/tushare. Accessed 1 July 2019.

[44] Wang X, Lin W. Stock market prediction using neural networks: does trading volume help in short-term prediction?. n.d.

[45] Weng B, Lu L, Wang X, Megahed FM, Martinez W (2018). Predicting short-term stock prices using ensemble methods and online data sources. Expert Syst Appl.

[46] Zhang S. Architectural complexity measures of recurrent neural networks, (NIPS). 2016. pp. 1–9.

[47] Zubair M, Fazal A, Fazal R, Kundi M (2019). Development of stock market trend prediction system using multiple regression. Computational and mathematical organization theory.

